# Beyond vision: Exploring the impact of visual perception on participation in children with autism spectrum disorder

**DOI:** 10.1371/journal.pone.0330457

**Published:** 2025-08-29

**Authors:** Sümeyye Belhan Çelik, Esma Özkan

**Affiliations:** 1 Occupational Therapy, Faculty of Hamidiye Health Sciences, University of Health Sciences, Istanbul, Türkiye; 2 Occupational Therapy, Faculty of Gulhane Health Sciences, University of Health Sciences, Ankara, Türkiye; Neighborhood Physical Therapy, UNITED STATES OF AMERICA

## Abstract

Visual perception plays a crucial role in the daily participation of children with Autism Spectrum Disorder (ASD) in everyday activities. Exploring the relationship between visual perception skills and participation levels can provide valuable insights into effective intervention strategies to enhance engagement in various settings. This study aimed to evaluate visual perception and participation levels in children with ASD in terms of demographic variables and to examine the relationships between visual perception skills and participation in daily life activities. Sixty-one children with autism (mean age = 8.21 ± 1.05 years) enrolled in a special education center were assessed using the Motor-Free Visual Perception Test – 4 (MVPT-4) for visual perception and the Child and Adolescent Scale of Participation (CASP) for participation levels across home, school, neighborhood, and community settings. Statistical analyses included correlation and regression analyses to examine relationships between variables. Findings indicated that boys participated more in home and school activities, whereas girls were more engaged in community settings. Additionally, children from nuclear families had higher participation levels than those from separated families. Regression analysis indicated that visual perception was strongly associated with participation levels (β = 0.617, p < 0.001), accounting for 55.8% of the variance in CASP Total scores. A significant positive correlation was found between visual perception and participation in home (r = 0.358, p < 0.001), school (r = 0.313, p = 0.014), and community activities (r = 0.361, p = 0.004), suggesting that better visual perception is linked to higher participation levels. The results suggest that visual perception is a significant factor influencing participation levels in children with ASD. Furthermore, family type showed a significant contribution to participation variance in the regression analysis. These findings underscore the importance of incorporating visual perception-based interventions to enhance the participation of children with ASD in everyday activities, yet they should be interpreted as correlational rather than causal, highlighting the need for future longitudinal or interventional research.

## Introduction

Autism Spectrum Disorder (ASD) represents a multifaceted neurodevelopmental condition that manifests early in childhood, significantly influencing an individual’s cognitive, emotional, and social development [1]. ASD is characterized by a constellation of behavioral features that include marked deficits in social communication, encompassing both verbal and nonverbal domains, and atypical patterns of social interaction that pervade multiple environments and contexts [2]. The increasing prevalence of ASD places considerable strain on healthcare systems, educational institutions, and social service agencies. To address these challenges, comprehensive and coordinated strategies are needed to effectively support individuals with ASD and their families. Furthermore, these efforts require the implementation of early detection and intervention programs. Ongoing research is also essential to better understand the underlying etiology, pathogenesis, and optimal treatment approaches for ASD [3]. One crucial aspect of ASD that has gained increasing attention is sensory processing differences. Sensory processing plays a fundamental role in how individuals perceive and respond to their environment, influencing their ability to interact with others and navigate daily life. Atypical sensory experiences, particularly in visual perception, are commonly reported in individuals with ASD. Among these, visual perception differences have been identified as particularly significant, affecting various aspects of cognition, behavior, and social interaction [1,4,5].

Visual perception is an important part of sensory processing that involves receiving, interpreting, and understanding visual information from the environment. This complex process uses coordinated brain systems to enable people to recognize objects, navigate spaces, and understand social cues [2]. There is a complex connection between visual perception and other cognitive functions. This connection demonstrates how crucial visual processing is in shaping a person’s development and how issues related to visual perception can affect social, motor, and cognitive functioning.

Visual perception is a fundamental cognitive skill that allows individuals to analyze and interpret visual information from their surroundings [6]. In individuals with ASD, these components may be affected at varying levels. Particularly, weaknesses in visual attention and discrimination skills can make it challenging to organize and interpret environmental stimuli. Difficulties in spatial perception and figure-ground differentiation may also lead to problems in recognizing objects and facial expressions [7]. These challenges contribute to difficulties in daily life activities and social interactions. For example, deficits in understanding the relationships between objects and integrating this information into daily activities can negatively impact an individual’s ability to use environmental cues effectively. It is well established that children with ASD struggle with processing and utilizing environmental information, especially in organizing and interpreting visual stimuli [8].

Visual perception in children with ASD often differs from neurotypical patterns in terms of processing methods and sensitivities. In ASD, there may be greater sensitivity to visual stimuli such as bright lights, contrasting colors, and complex patterns, which can lead to sensory overload and discomfort. However, there may be less sensitivity to subtle visual features such as facial expressions and body language, which negatively affects the ability of children with ASD to interpret social cues [9,10]. Children with autism spectrum disorder often exhibit atypical gaze patterns, such as reduced eye contact, a tendency to focus on details rather than the whole image, and difficulties in tracking moving objects. Laycock et al. (2020) reported that individuals with ASD exhibit inefficiencies in processing fast and dynamic visual information, which can result in delays in social interactions [11]. Another study found that children with ASD have lower visual perception skills compared to typically developing children, which in turn negatively affects their participation in daily life activities [12].

Visual processing differences can affect how children with ASD perceive their environment, process information, and engage with others. These difficulties may lead to challenges in interpreting social cues, navigating complex environments, and maintaining attention—especially when visual scenes are cluttered with irrelevant details—thereby hindering participation in daily activities [2,5]. This particularly affects the ability to understand and respond to visual information in school, online environments, and daily life [13]. Visual motion processing issues can make it difficult to track moving objects, predict spatial trajectories, and perform dynamic visual processing tasks. These various visual processing challenges contribute to the multifaceted nature of ASD and highlight the importance of understanding and address these challenges to improve the adaptive functioning and quality of life of children with ASD [2,14]. The integrity of visual processing significantly affects how individuals with ASD perceive their environment and interact with it, impacting their participation in daily activities and social interactions [4].

Understanding and addressing the impact of visual perception on the lives of children with ASD is crucial to developing targeted interventions that promote inclusion and improve their overall well-being. However, while previous studies have described visual processing anomalies in ASD, there remains a limited understanding of how these visual perception difficulties specifically influence everyday participation in various life contexts. This study addresses this gap by examining the direct relationship between visual perception skills and participation levels, considering the influence of demographic variables, to guide targeted interventions. This study aimed to evaluate visual perception and participation levels in children with ASD, considering demographic variables, and to examine the relationship between visual perception skills and daily life participation. 

## Materials and methods

### Design

This descriptive and cross-sectional study examines the relationship between the total score of the “Motor-Free Visual Perception Test – 4” (MVPT-4), which assesses visual perception in children with Autism Spectrum Disorder, and the total score of the “Child and Adolescent Scale of Participation” (CASP), which evaluates participation in home, school, neighborhood, and community settings. The study was conducted in accordance with the STROBE statement, which provides guidelines for observational research. The study protocol was approved by the Hamidiye Non-invasive Investigation Ethics Committee (13 February 2023-22/625).

The simple random sampling method was used to select the participants in the study. Before administering the data collection forms, verbal consent was obtained from the participating children, and written informed consent was acquired from their parents through the “Parent Consent Form.” After receiving consent, the researchers conducted questionnaires with the parents of children diagnosed with ASD. The survey forms were printed and administered face-to-face.

### Sample

Between 15 June 2023 and 22 July 2024, a total of 79 children were interviewed for this study, and 18 children did not meet the inclusion criteria. The population of this study consisted of 61 children diagnosed with ASD who were receiving education at the Special Education and Rehabilitation Center in Istanbul. The sample size was calculated using G*Power Version 3.1.9.4 software for Pearson correlation analysis. Setting the Type I error rate at 0.05 and the statistical power at 0.80, with the effect size index for Pearson correlation coefficient set at medium (0.35), as suggested by Cohen. The required sample size was determined to be 61 participants. The inclusion criteria for the study are as follows: having a diagnosis of ASD, being between the ages of 7 and 10, the voluntary participation of both the child and their family, having a basic level of communication skills (e.g., understanding simple instructions), regularly attending education at a special education and rehabilitation center. The exclusion criteria include having a known neurological disorder or cortical visual impairment, having a secondary diagnosis (e.g., Down syndrome, intellectual disability, or Attention Deficit Hyperactivity Disorder), and the use of visual or auditory assistive devices (e.g., glasses or hearing aids).

### Data collection tools

In this study, data were collected through the Personal Information Form, the Child and Adolescent Scale of Participation (CASP), and the Motor-Free Visual Perception Test-4 (MVPT-4).

#### The sociodemographic information form.

It included sociodemographic information such as age, gender, number of siblings, school grade, family educational and income levels, caregiver’s employment status, and family structure.

#### Motor-Free Visual Perception Test-4 (MVPT-4).

MVPT-4 is a normative test developed by Colarusso and Hammill in 1996 as a revision of MVPT-3, designed to assess visual perception skills quickly, reliably, and comprehensively. It is applicable for individuals aged 4 to 80 + years [15]. The test consists of 45 questions evaluating five main components of visual perception: spatial relations, visual discrimination, figure-ground, visual closure, and visual memory. Each correct answer is scored as 1 point, while incorrect or unanswered questions receive a score of 0. The total score is calculated by summing the number of correct responses. The test is administered by presenting black figures on a white background, and participants are required to select the correct answer from multiple-choice options. The administration time is approximately 20–30 minutes. The MVPT-4 has demonstrated good validity and reliability (Cronbach’s α: 0.70–0.87) [16,17].

#### Child and Adolescent Scale of Participation (CASP).

CASP assesses the participation of children aged five and older in various settings, including home, school, neighborhood, and community. Covering a broad spectrum of daily life activities, CASP consists of 20 items categorized into four subscales: Home Participation (4 items), School Participation (5 items), Neighborhood and Community Participation (4 items), and Home and Community Living Activities (5 items). The questionnaire is completed by parents or caregivers, who rate each itemon a 0–4 scale. The total score, ranging from 0 to 100, reflects the child’s level of participation, with higher scores indicating greater participation [18]. The Turkish adaptation of CASP was carried out by Atasavun Uysal et al., and its validity and reliability have been confirmed [18,19].

### Statistical analyses

All data were presented as mean ± standard deviation (SD) or relevant statistical measures, and statistical significance was set at **p* *< 0.05. As descriptive statistics, mean and standard deviation (mean±SD) were used for continuous data, and frequency and percentage were used for categorical data.

The distribution of continuous variables that follow a normal distribution between two groups was tested using the Student’s t test, while those not following a normal distribution were analyzed using the Mann-Whitney U test. For continuous variables across three or more groups, the One-Way ANOVA was used for normally distributed data, whereas the Kruskal-Wallis test was applied for non-normally distributed data. To determine the source of significant differences, the Bonferroni test was used for multiple comparisons following One-Way ANOVA, and the Dunn-Bonferroni test was employed after the Kruskal-Wallis test.

A Stepwise Multiple Linear Regression analysis was performed to develop a predictive model for the CASP Total variable. The assumptions of linear regression were evaluated as follows: normality was assessed using the Kolmogorov-Smirnov test, and linearity was checked with scatter plots. To evaluate model adequacy, autocorrelation of residuals was evaluated using the Durbin-Watson statistic, and multicollinearity was assessed with the Variance Inflation Factor (VIF). Influential and distant observations were examined using Cook’s Distance and Covariance Ratio, while excessively outlier observations and homoscedasticity (variance homogeneity) were evaluated with standardized residual plots. The normality of residuals was confirmed using the Kolmogorov-Smirnov test of standardized residuals [20]. Data were analyzed using IBM SPSS 21 (IBM SPSS Inc., Chicago, IL), and the significance level was set at 0.05.

## Results

The study included 61 children with autism (mean age = 8.21 ± 1.05 years). Of the participants, 37 (60.70%) were boys and 24 (39.30%) were girls. With respect to number of siblings, 20 (32.79%) were only children, 22 (36.07%) had one sibling, 13 (21.31%) had two siblings, and 6 (9.84%) had three siblings. In terms of grade level, 19 (31.15%) were in the first grade, 24 (39.34%) in the second grade, 12 (19.67%) in the third grade, and 6 (9.84%) in the fourth grade. For family income, 11 (18.03%) reported expenses exceeding income, 32 (52.46%) reported income equal to expenses, and 18 (29.51%) reported income exceeding expenses. The educational level of parents was as follows: 9 (14.75%) had primary, 22 (36.07%) secondary, 15 (24.59%) high school, and 15 (24.59%) university education. The employment status of caregivers showed that 26 (42.62%) were employed, while 35 (57.38%) were unemployed. In terms of family type, 44 (72.13%) were from nuclear families, 7 (11.48%) from extended families, and 10 (16.39%) from divorced families.

The distributions of MVPT Total, CASP Total, CASP Home Participation, CASP Neighborhood and Community Participation, CASP School Participation, and CASP Home and Community Living Activities scores across socio-demographic groups were compared, and the results are presented in Table 1.

**Table 1:** Distribution of MVPTand CASP scores by socio-demographic variables.

N = 61		MVPT TOTAL Mean ± SD	CASP TOTAL Mean ± SD	CASP HOME Mean ± SD	CASP NEIGHBORHOOD AND COMMUNITY Mean ± SD	CASP SCHOOL Mean ± SD	CASP HOME COMMUNITY LIVING Mean ± SD
**Age (years)**	**7 (n = 18)**	28.33 ± 7.62	53.28 ± 7.09	19.33 ± 2.95	10.06 ± 3.13	15.17 ± 2.48	8.72 ± 3.49
	**8 (n = 22)**	27.55 ± 7.35	54.91 ± 6.49	20.72 ± 2.69	10.23 ± 2.54	14.73 ± 3.18	9.36 ± 4.20
	**9 (n = 11)**	26.91 ± 6.88	54.10 ± 7.85	19.54 ± 2.81	10.09 ± 2.55	14.73 ± 2.97	9.73 ± 4.00
	**10 (n = 10)**	27.80 ± 8.35	54.90 ± 8.02	19.80 ± 2.10	9.50 ± 3.17	14.50 ± 2.80	11.10 ± 2.77
**Test Statistic**		χ2 = .211^*^	F(3.57)=.201^**^	χ2 = 1.613	F(3.57)=.154	F(3.57)=.139	F(3.57)=.880
**p**		.976	.895	.657	.927	.936	.457
**Gender**	**Boy (n = 37)**	27.59 ± 7.28	54.14 ± 7.74	20.62 ± 2.71	9.57 ± 2.97	15.59 ± 2.78	8.43 ± 3.86
	**Girl (n = 24)**	27.87 ± 7.61	54.50 ± 5.93	18.92 ± 2.41	10.75 ± 2.33	13.63 ± 2.50	11.21 ± 2.93
**Test Statistic**		t = .144^***^	t = .196	Z = 2.660^****^	Z = 1.655	t = 2.808	t = 3.005
**p**		.886	.845	.008	.098	.007	.004
**Number of Siblings**	**0 (n = 20)**	28.45 ± 7.60	53.85 ± 7.16	19.75 ± 2.88	9.20 ± 2.63	14.50 ± 2.01	10.55 ± 3.56
	**1 (n = 22)**	27.14 ± 7.70	55.18 ± 7.00	19.77 ± 2.77	11.23 ± 2.39	14.59 ± 3.59	9.59 ± 3.55
	**2 (n = 13)**	26.08 ± 5.91	52.31 ± 6.39	20.15 ± 3.02	9.31 ± 2.72	14.85 ± 2.76	8.00 ± 3.56
	**3 (n = 6)**	30.83 ± 8.54	56.67 ± 8.50	20.83 ± 0.98	10.0 ± 3.74	16.67 ± 1.75	9.17 ± 5.19
**Test Statistic**		χ2 = 1.581^*^	F(3.57)=.707	χ2 = .829^*^	F(3.57)=2.415	χ2 = 3.625^*^	F(3.57)=1.248
**p**		.664	.552	.843	.076	.305	.301
**Grade level**	**1 (n = 19)**	27.89 ± 7.73	53.42 ± 6.57	19.32 ± 2.87	10.37 ± 2.79	15.26 ± 2.31	8.47 ± 3.56
	**2 (n = 24)**	27.63 ± 7.29	55.50 ± 6.62	20.58 ± 2.81	10.13 ± 2.89	14.83 ± 3.26	10.08 ± 3.90
	**3 (n = 12)**	28.58 ± 6.84	53.50 ± 8.53	19.67 ± 2.71	9.75 ± 2.73	14.33 ± 2.53	9.75 ± 4.09
	**4 (n = 6)**	25.67 ± 8.82	53.67 ± 7.99	20.00 ± 1.55	9.17 ± 2.79	14.33 ± 3.50	10.17 ± 3.19
**Test Statistic**		χ2 = .944^*^	χ2 = 1.540^*^	χ2 = 1.653^*^	χ2 = .923^*^	F(3.57)=.323	F(3.57)=.737
**p**		.815	.673	.647	.820	.809	.534
**Income Level**	**Income<Expense (n = 11)**	26.73 ± 8.37	53.19 ± 6.88	19.82 ± 2.14	9.82 ± 2.36	14.64 ± 1.50	8.91 ± 3.81
	**Income = Expense** **(n = 32)**	27.38 ± 6.65	53.41 ± 6.48	19.84 ± 2.73	9.91 ± 2.80	14.72 ± 3.03	9.03 ± 3.93
	**Income>Expense** **(n = 18)**	28.89 ± 8.15	56.50 ± 7.91	20.22 ± 3.10	10.39 ± 3.07	15.11 ± 3.16	10.78 ± 3.26
**Test Statistic**		χ2 = .772^*^	χ2 = 2.264^*^	χ2 = 1.138^*^	F(2.58)=.209	χ2 = .614^*^	F(2.58)=1.449
**p**		.680	.322	.566	.812	.735	.243
**Parental Education Level**	**Primary School (n = 9)**	25.67 ± 6.61	54.22 ± 6.55	18.33 ± 3.20	10.67 ± 2.34	13.78 ± 2.44	11.44 ± 2.65
	**Middle School (n = 22)**	28.68 ± 8.48	53.77 ± 7.34	20.09 ± 2.18	10.00 ± 2.37	14.36 ± 2.59	9.45 ± 4.27
	**High School (n = 15)**	27.73 ± 7.37	52.87 ± 6.25	19.60 ± 3.20	9.33 ± 2.94	15.20 ± 2.62	8.73 ± 3.81
	**University (n = 15)**	27.47 ± 6.32	56.47 ± 7.72	21.07 ± 2.25	10.40 ± 3.44	15.73 ± 3.43	9.27 ± 3.37
**Test Statistic**		F(3.57)=.354	F(3.57)=.714	χ2 = 7.770^*^	F(3.57)=.550	χ2 = 3.936^*^	F(3.57)=1.035
**p**		.786	.548	.051	.650	.268	.384
**Caregiver’s Employment Status**	**Employed (n = 26)**	26.50 ± 7.01	54.62 ± 6.58	20.15 ± 2.88	10.04 ± 2.70	14.96 ± 3.36	9.46 ± 3.84
	**Unemployed (n = 35)**	28.60 ± 7.56	54.03 ± 7.43	19.80 ± 2.61	10.03 ± 2.86	14.71 ± 2.40	9.57 ± 3.74
**Test Statistic**		Z = 1.022	Z =.088	Z = .148	t = .014	Z = .309	t = .112
**p**		.307	.930	.883	.989	.758	.911
**Family Type**	**Nuclear Family (n = 44)**	28.73 ± 7.46	55.57 ± 7.06	20.61 ± 2.44	10.29 ± 2.84	15.55 ± 2.67	9.18 ± 3.91
	**Extented Family (n = 7)**	29.71 ± 7,34	54.00 ± 7.44	19.14 ± 2.48	10.43 ± 2.23	14.00 ± 1.00	10.43 ± 4.47
	**Divorced Family (n = 10)**	21.80 ± 3.29	48.80 ± 3.52	17.60 ± 2.76	8.60 ± 2.59	12.20 ± 2.78	10.40 ± 2.32
**Test Statistic**		F(2.58)=4.356	F(2.58)=4.186	χ2 = 10.203	χ2 = 3.159	χ2 = 12.348	F(2.58)=.651
**p**		.017	.020	.006	.206	.002	.525

MVPT: Motor-Free Visual Perception Test. CASP: Child and Adolescent Scale of Participation

* Kruskal Wallis test statistic value.

** One-way ANOVA test statistic value.

*** Student’s t test statistic value.

**** Mann-Whitney U test statistic value. Statistical significance p <  .05.

When evaluating the distributions of MVPT Total, CASP Total, and allsub-dimensions across various age groups, it was determined that no statistically significant differences existed, with the means across groups being comparable (p =  .976, p =  .895, p =  .657, p =  .927, p =  .936, and p =  .457), according to Table 1.

Comparative analysis of the MVPT Total, CASP Total, and CASP Neighborhood and Community Participation scale score distributions across gender groups revealed similarities in the average scores of male and female participants (p =  .886, p =  .845, and p =  .098, respectively). The averages of CASP Home Participation and CASP School Participation were statistically significantly higher in male participants than in female participants (p =  .008 and p =  .007), whereas the average of the CASP Home Community Activities variable was significantly higher in female participants than in male participants (p =  .004).

Upon assessing the distributions of total MVPT, total CASP, and all CASP sub-dimensions in relation to the number of siblings, it was determined that no statistically significant differences were present, with comparable means across groups (p =  .664, p =  .552, p =  .843, p =  .076, p =  .305, and p =  .301, respectively).

After analyzing the distributions of MVPT Total, CASP Total, and all CASP sub-dimensions across various grade groups, it was determined that no statistically significant differences existed, and the group means were comparable (p =  .815, p =  .673, p =  .647, p =  .820, p =  .809, and p =  .534, respectively).

When assessing the distributions of MVPT Total, CASP Total, and all CASP sub-dimensions across income status groups, it was found that no statistically significant differences existed. Participants with incomes surpassing their expenses exhibited higher average scale scores than the other groups; however, this distinction lacked statistical significance (p =  .680, p =  .322, p =  .566, p =  .812, p =  .735, and p =  .243).

Upon examining the distributions of MVPT Total, CASP Total, and all CASP sub-dimensions across the Family Education Level categories, it was determined that no statistically significant differences existed, with the group means being comparable (p =  .786, p =  .548, p =  .051, p =  .650, p =  .268, and p =  .384).

Upon assessing the distributions of MVPT Total, CASP Total, and all CASP sub-dimensions across the Caregiver Employment Status groups, it was determined that no statistically significant differences were present, with the means across the groups being comparable (p =  .307, p =  .930, p =  .883, p =  .989, p = .758, and p =  .911).

A statistically significant difference was observed in the distributions of MVPT Total, CASP Total, CASP Home Participation, and CASP School Participation scale scores among Family Type groups.Post-hoc Bonferroni and Dunn-Bonferroni test revealed that the average scale scores for MVPT Total, CASP Total, CASP Home Participation, and CASP School Participation were statistically significantly lower in participants from Separated Families compared to those from Nuclear Families and Extended Families (p = .017, p = .020, p = .006, and p = .002, respectively). The averages for CASP Neighborhood and Community Participation activities were comparable among participants (p = .206 and p = .525).

### Correlation analysis

The relationship between visual perception and participation and its sub-dimensions were evaluated using Spearman correlation analyses in Table 2. The results showed that there is a significant relationship between children’s visual perception levels and their participation in activities (p<0.05).

**Table 2:** The relationship analyses between scales and their subdimensions.

		MVPT TOTAL	CASP TOTAL	CASP HOME	CASP NEIGHBORHOOD AND COMMUNITY	CASP SCHOOL	CASP HOME COMMUNITY LIVING
MVPT TOTAL	rho	1	.611	.358	.491	.313	.361
	p		**< .001**	**< .001**	**< .001**	**.014**	**.004**
CASP TOTAL	rho		1	.511	.659	.648	.558
	p			**< .001**	**< .001**	**< .001**	**< .001**
CASP HOME	rho			1	.077	.499	−.133
	p				.554	**< .001**	.308
CASP NEIGHBORHOOD AND COMMUNITY	rho				1	.263	.365
	p					**.040**	**.004**
CASP SCHOOL	rho					1	.361
	p						**.004**

MVPT: Motor-Free Visual Perception Test. CASP: Child and Adolescent Scale of Participation. rho: Spearman correlation coefficient. Statistical significance level (p <  .05).

The results of the multivariate linear regression analysis, which was conducted in two stages using the Ordinary Least Squares method for parameter estimation with the Stepwise variable selection method, are presented in Table 3. Two variables, MVPT Total and Family Type, were used in the study. A correlation analysis showed that these variables were statistically related to CASP Total. Given that the Family type variable is qualitative, it was incorporated into the analysis as a dummy variable. The reference category was the Separated Family category, and the indicator coding method was employed.

**Table 3:** Multivariable linear regression analysis of factors affecting total CASP.

	β	SE(β)	BETA	t	p	VIF	R^2^	F
**First Step**								
Intercept	36.542	2.403		15.204	< .001	1.000	.517	F(1.57)=60.993 p < .001
MVPT TOTAL	.658	.084	.719	7.810	< .001			
**Second Step**								
Intercept	35.468	2.366		14.993	< .001		.558	F(2.56)=35.390 p < .001
MVPT TOTAL	.617	.083	.674	7.414	< .001	1.048		
Nuclear Family	3.095	1.352	.208	2.290	.026	1.048		

MVPT: Motor-Free Visual Perception Test, β: Beta, SE(β): Standard Error of Beta, BETA: Standardized Coefficient, VIF: Variance Inflation Factor, R²: Coefficient of Determination, t: t statistic value, F: F-statistic value. Statistical significance level (p <  .05).

The errors were normally distributed, and there was no issue of changing variance (heteroscedasticity) as a result of examining the standardized residual plots of the obtained model. The Durbin-Watson test (D-W = 1.823) also revealed that the errors do not exhibit any autocorrelation.

We excluded two extremely distant and influential observations from the dataset during the observation analysis, also known as the case analysis. We got a statistically significant model from the multiple linear regression analysis of the last 59 observations in the dataset (F(2,56)=35.390, p <  .001). Multicollinearity was not detected (VIF < 10) [21].

The MVPT Total and Core Family variables were significant contributors in the model that explains the changes in the CASP Total score (p <  .001 and p =  .026, respectively).

The MVPT Total and Core Family variables are significant predictors. When the MVPT Total variable goes up by one unit, the CASP Total score goes up by .617 units. On the other hand, when the Core Family variable goes up, the prediction model goes up by 3.095 units compared to the reference category of the separated family category.

The independent variable with the greatest influence on the model was assessed using the standardized regression coefficients (BETA) among the predictors in it. The variable MVPT Total has the greatest contribution to the model, as evidenced by its standardized regression coefficient of .674. There are two separate factors in the model that explain 55.80% of the change in the CASP Total score These are MVPT Total (.617) and Nuclear Family (3.095) (Table 3).

## Discussion

This study investigated visual perception and participation levels in relation to demographic factors in children with autism spectrum disorder (ASD) and examined the correlation between children’s visual perception abilities and their participation in daily activities. Analysis of sociodemographic variables showed that male children had significantly higher participation in home and school settings, while female children showed higher participation in community settings. The relationship between visual perception and participation in our study showed that children with higher visual perception skills also had higher participation levels in daily life activities. In addition, significant differences in visual perception and participation were found in terms of family type. Since the data represent a single time point, these results indicate an association rather than a confirmed cause-and-effect link. It is also possible that other unmeasured factors, such as general cognitive capacity or family support, may influence both visual perception and participation. Therefore, future longitudinal or interventional studies that control for these potential confounders are recommended to clarify this relationship.

Although visual perception showed a significant association with participation, its interaction with demographic variables warrants consideration. Our study found that factors such as age, grade level, family income, caregiver employment status, family education, and number of siblings did not significantly affect visual perception or participation scores, aligning with previous research suggesting that demographic factors like age and income have limited impact and that participation is more closely related to social skills and contextual support [22]. Regarding siblings, while having more siblings may increase opportunities for social interaction and problem-solving [23,24], some studies suggest that being an only child may foster better emotional regulation and communication due to more focused parental attention [25,26]. **In our sample, however, sibling number was not a significant factor, suggesting that family dynamics and parental support play a more critical role** [27]. These findings indicate that sibling quantity and other demographic factors alone are insufficient to explain participation outcomes and should be interpreted in the context of broader family dynamics and psychosocial factors.

Consistent with prior research, our results showed that demographic characteristics such as age, income, parental education, and sibling number did not significantly affect visual perception or participation levels [12,22,28]. Our study revealed that male participants scored considerably higher in Home Participation and School Participation, but female participants exhibited better scores in Home Community Activities. This pattern aligns with previous findings indicating that boys are generally more engaged in physical and structured activities, whereas girls participate more in social and community contexts [11]. These differences likely reflect gender roles and cultural expectations in Türkiye and highlight the need for cross-cultural comparisons in future studies.

In addition to gender, family structure emerged as a significant factor influencing both visual perception and participation. Children from fractured families had lower MVPT and CASP scores than those from nuclear or extended families (p < 0.05). Prior studies similarly suggest that children from intact families benefit from stronger social support and fewer emotional and financial difficulties, which may enhance participation outcomes [29–33]. Our regression analysis confirmed that family structure is an important contextual factor and should be considered together with psychosocial support when developing intervention plans for children with autism. These findings highlight the need for therapists, teachers, and caregivers to consider family context when planning interventions. Given that family structure was found to influence both visual perception and participation, children from fractured families may benefit from tailored visual perception support and additional social and emotional resources to enhance their engagement. Incorporating visual perception-focused activities into daily routines and educational settings can help improve participation across various life domains for children with ASD.

Sensory input processing dysfunction is a well-known challenge in individuals with autism and contributes to difficulties in organizing environmental stimuli. Research shows that abnormalities in brain regions such as the occipital and parietal lobes, as well as dysfunction in the magnocellular pathway, may affect visual attention and social cognitive abilities [34–36]. These neurobiological factors can help explain why some children with autism struggle with perceiving dynamic social cues and maintaining engagement in social contexts. Our findings showed that lower visual perception scores were associated with lower participation in everyday life activities, which may reflect challenges in perceiving facial emotions, gestures, and eye contact [36].

Our study reveals significant relationships between different participation domains and visual perception abilities. The notable correlation between CASP Home Participation and MVPT-4 suggests that the autonomy of children with autism at home is strongly linked to their visual perception abilities. This indicates that visual perception is essential for self-care activities, indoor play, and the upkeep of everyday routines at home. The relationship between Neighborhood and Community Participation and visual perception underscores the significance of visual perception in the adaptation of children with autism to their external environment. Nonetheless, the results of our study do not indicate a direct causal relationship. Despite the identification of a correlation, the nature of this association remains ambiguous. Although advanced visual perception abilities may facilitate social engagement, individuals with greater social experience may also enhance their visual perception with time. Consequently, future studies should examine the direction and causality of this relationship in more detail using longitudinal designs.

The development of visual perception skills in children with autism may also be affected by differences in cultural and educational systems. Jasmin et al. (2009) suggested that delays in self-care may relate not only to individual development but also to family dynamics and cultural norms [33]. Differences in parental roles across cultures can also affect how independence skills develop; for example, Western families often encourage autonomy, whereas some Asian families may provide more direct care [37]. Given the significant association found between CASP Home Participation and visual perception in this study, future research should further examine how cultural context influences this relationship.

The significant relationship found between School Participation and Visual Perception in our study reveals the impact of visual perception on academic life. Mitchell ve Ropar (2004) asserted that while children with autism may excel in certain visual perception abilities, they often struggle with the holistic visual organization skills necessary for academic tasks [38]. Due to the reliance of academic skills, including reading, writing, following the board, and graph interpretation, on visual perception processes, children with autism who experience visual perception issues are likely to encounter challenges in academic participation [7,39].

Visual perception skills and motor capabilities are known to be interconnected. Vatreyan et al. (2014) highlighted a significant relationship between the academic functioning of children with autism and their visual perception skills, particularly underscoring the essential role of visual-motor integration in academic and social participation [12]. Interventions such as cognitive-based visual perception therapy, visual-motor integration training, and technology-assisted methods may enhance the functionality of individuals with autism in academic and social domains.

The physical and sensory arrangements of educational environments can directly influence the visual perception processes of children with autism. It is emphasized that sensory factors such as light, color, texture and spatial organization should be arranged by taking into account individual differences [40]. Supporting visual perception through environmental adjustments and sensory integration strategies implemented by occupational therapists may effectively augment academic and social participation.

Furthermore, project-based learning processes can allow children to explore their environment, observe and analyze objects, and express this knowledge through drawing, modeling, and various creative methods [40]. These procedures may be more efficacious when integrated with structured educational programs and teaching techniques specifically designed to enhance reading and writing skills. Educational approaches that particularly address visual perception, such as the Frostig Developmental Visual Perception Program, may substantially improve the academic success and daily living skills of children with autism [41].

In addition to structured educational programs that support visual perception, cognitive mechanisms such as attention and executive functions also play a crucial role in the participation and learning processes of children with ASD. These cognitive processes are closely linked to visual perception and can influence academic performance, social interactions, and independent daily living skills.

Visual perception is tightly associated with cognitive mechanisms such as attention and executive functions. In a study conducted by May et al. (2003), it was found that attention switching difficulties can negatively affect academic and daily life skills in children with autism [42]. Future studies should investigate how visual perception underpins processes such as independent movement, social interaction, and self-care, particularly when attention and executive functions are directly linked to participation. This investigation should incorporate confounding variables, including family dynamics, cognitive learning processes, and learning styles, in greater detail. Moreover, while designing treatments to enhance involvement, it is essential to acknowledge the individual differences in visual perception and cognitive learning processes among individuals with autism; hence, interventions should be tailored to each individual.

This study highlights the unique visual perceptual challenges faced by children with ASD and their impact on home, school, and community participation. While much of the existing literature examines visual perception in conjunction with motor skills [43], our study contributes to the field by evaluating visual perception independently using the MVPT-4 assessment tool. By considering participation beyond the academic context and encompassing multiple life domains, our findings provide a more comprehensive understanding of how visual perception influences various aspects of daily life.

To enhance the effectiveness of interventions that support visual perception and assess their long-term impact on participation, further research is needed. Specifically, longitudinal studies are essential to establish causal relationships between visual perception and independent living skills. Moreover, future experimental studies should examine the long-term effects of interventions aimed at improving visual perception on broader social and community participation, rather than solely focusing on academic and individual skill development. An integrated treatment approach combining visual perception training with social skills, motor skills, physical therapy, and family support may further enhance participation outcomes and offer a more comprehensive framework for intervention [44]. In this regard, research comparing the effectiveness of different intervention approaches—such as sensory integration training, cognitive-based visual perception therapies, and technology-assisted methods—would be highly valuable. In particular, studies exploring the impact of technology-based visual perception training programs on children with ASD could offer significant insights into their potential to support both academic achievement and daily life skills [45]. In particular, therapists can also use standardized visual perception assessments such as the MVPT-4 as a screening tool to identify children who may benefit from early intervention targeting visual-perceptual skills to enhance participation. Additionally, the findings of this study can guide therapists, teachers, and caregivers in creating supportive learning environments, implementing effective visual aids, and adapting activities to foster greater participation and engagement.

### Limitations

This study has some limitations that should be considered when interpreting the findings. First, it focuses solely on visual perception, without assessing the impact of other sensory processing domains (e.g., auditory or tactile processing) on participation. Future research should compare the influence of different sensory modalities on participation to provide a more comprehensive perspective. Additionally, family-related factors such as parental involvement, socio-economic status, and cultural expectations may influence both visual perception and participation. Although our study accounted for family structure, other environmental and familial variables should be further examined to better understand their role in shaping participation outcomes.

Second, while significant correlations were found between visual perception and participation levels, this study does not establish a causal relationship. It remains unclear whether stronger visual perception skills promote higher participation levels or whether increased social experiences contribute to the development of visual perception abilities over time. Therefore, future studies should employ longitudinal designs to further investigate the directionality and causality of this relationship.

Lastly, to achieve greater sample homogeneity, children with comorbid conditions or assistive device use were excluded; however, this methodological choice may limit the generalizability of the findings and reflects an inherent trade-off between internal consistency and external validity.

In addition, the study was conducted with a relatively small sample of children enrolled in a single special education center in Türkiye, which may limit the generalizability of the findings. Future research should validate these results in larger, more diverse samples from multiple centers and varied educational and cultural contexts to ensure broader applicability.

## Supporting information

S1 Data Dataset. (XLSX)
